# Data report on inflammatory C–C chemokines among insulin-using women with diabetes mellitus and breast cancer

**DOI:** 10.1016/j.dib.2017.02.045

**Published:** 2017-02-22

**Authors:** Zachary A.P. Wintrob, Jeffrey P. Hammel, George K. Nimako, Zahra S. Fayazi, Dan P. Gaile, Erin E. Davis, Alan Forrest, Alice C. Ceacareanu

**Affiliations:** a State University of New York at Buffalo, Dept. of Pharmacy Practice, NYS Center of Excellence in Bioinformatics and Life Sciences, 701 Ellicott Street, Buffalo, NY 14203, United States; b Cleveland Clinic, Dept. of Biostatistics and Epidemiology, 9500 Euclid Ave., Cleveland, OH 44195, United States; c State University of New York at Buffalo, Dept. of Biostatistics, 718 Kimball Tower, Buffalo, NY 14214, United States; d The UNC Eshelman School of Pharmacy, Division of Pharmacotherapy and Experimental Therapeutics, Campus Box 7569, Chapel Hill, NC 27599, United States; e Roswell Park Cancer Institute, Dept. of Pharmacy Services, Elm & Carlton Streets, Buffalo, NY 14263, United States

**Keywords:** Inflammation, Inflammatory cytokines, C-C chemokines, CCL-2, CCL-3, CCL-4, CCL-5, Insulin, Breast cancer, Diabetes, Tumor-associated macrophages, Cancer prognosis

## Abstract

Injectable insulin use may interfere with pro-inflammatory cytokines’ production and, thus, play a role in the activation of tumor-associated macrophages - a process mainly influenced by inflammatory C–C chemokines. The data presented shows the relationship between pre-existing use of injectable insulin in women diagnosed with breast cancer and type 2 diabetes mellitus, the inflammatory C–C chemokine profiles at the time of breast cancer diagnosis, and subsequent cancer outcomes. A Pearson correlation analysis stratified by insulin use and controls is also provided. We present the observed relationship between the investigated C–C chemokines and between each of these biomarkers and previously reported adipokines levels in this study population [1].

**Specifications Table**

**Table:** 

Subject area	Clinical and Translational Research
More specific subject area	Biomarker Research, Cancer Epidemiology
Type of data	Tables
How data was acquired	Tumor registry query was followed by vital status ascertainment, and medical records review
	Luminex®-based quantitation from plasma samples was conducted for the following pro-inflammatory C–C chemokines: Chemokine ligand 2, CCL-2 (monocyte chemoattractant protein 1, MCP-1); chemokine ligand 3, CCL-3 (macrophage inflammatory protein 1α, MIP-1α); chemokine ligand 4, CCL-4 (macrophage inflammatory protein 1β, MIP-1β); and chemokine ligand 5, CCL-5 (regulated on activation normal T cell expressed and secreted, RANTES).
	A Luminex®200^TM^ instrument with Xponent 3.1 software was used to acquire all data
Data format	Analyzed
Experimental factors	The above described pro-inflammatory C–C chemokines were determined from the corresponding plasma samples collected at the time of breast cancer diagnosis
Experimental features	According to a previously described study design, the dataset included 97 adult females with diabetes mellitus and newly diagnosed breast cancer (cases) and 194 matched controls (breast cancer only) [1]. Clinical and treatment history were evaluated in relationship with cancer outcomes and pro-inflammatory cytokine profiles. A biomarker correlation analysis was performed between the studied C-C chemokines and between each of them and the cytokine levels already reported elsewhere for this particular patient population [1], [2], [3], [4], [5], [6], [7], [8], [9]. The additional correlations were provided for completeness and usability of this data.
Data source location	United States, Buffalo, NY - 42° 53׳ 50.3592"N; 78° 52׳ 2.658"W
Data accessibility	The data is with this article

**Value of the data**

• Monocytes’ infiltration and their activation to tumor-associated macrophages upon recruitment into the tumor tissue is a crucial process for tumor growth and metastasis [3]. Their mobilization is a chemotactic response mediated by tumor-derived factors, among which the C–C chemokines CCL-2, 3, 4, and 5 [4], [5], [6], [7], [8], [9]
• The combined contribution of CCL-2, 3, 4, and 5 is responsible for the vast functionality of the macrophage phenotypes in response to changing environmental stimuli [4], [5], [6], [7], [8]
• This dataset represents the observed relationship between injectable insulin use, circulating pro-inflammatory C–C chemokines at breast cancer diagnosis and outcomes
• Reported data has the potential to guide future studies evaluating the impact of insulin-regulated signaling on activation of tumor-associated macrophages in breast cancer
• Our observations can assist further research clarifying the role of insulin in the regulation of the pro-inflammatory signaling leading to pro-tumorigenic activity in the breast tumor microenvironment

## 1 Data

Reported data represents the observed association between use of injectable insulin preceding breast cancer and the pro-inflammatory C–C chemokine profiles at the time of cancer diagnosis in women with diabetes mellitus (Table 1). Data in Table 2 includes the observed correlations between pro-inflammatory C–C chemokines stratified by type 2 diabetes mellitus pharmacotherapy and controls, as well as already reported biomarkers’ correlation with each of the studied C–C chemokines is presented in Table 2. The details regarding adiponectin, leptin, C-reactive protein, C-peptide, tumor necrosis factor α, interleukin 1β and its receptor antagonist, interleukin 6, and interleukin 10 determination from plasma, and their association with cancer outcomes and use of injectable insulin has been previously reported [1] or is reviewed under a separate dataset [2].

## 2 Experimental design, materials and methods

This work was completed following a previously described case-control study design [1]. Briefly, the evaluation of pro-inflammatory C-C chemokine profiles association with injectable insulin use and BC outcomes was carried out under two protocols approved by both Roswell Park Cancer Institute (EDR154409 and NHR009010) and the State University of New York at Buffalo (PHP0840409E). Demographic and clinical patient information was linked with cancer outcomes and biomarker profiles of corresponding plasma specimen harvested at BC diagnosis and banked in the Roswell Park Cancer Institute Data Bank and Bio-Repository.

### 2.1 Study population

All incident breast cancer cases diagnosed at Roswell Park Cancer Institute (01/01/2003-12/31/2009) were considered for inclusion (*n*=2194). Medical and pharmacotherapy history were used to determine the baseline presence of diabetes following the previously described method [1].

### 2.2 Inclusion and exclusion criteria

All adult women with pre-existing diabetes at breast cancer diagnosis having available banked treatment-naïve plasma specimens (blood collected prior to initiation of any cancer-related therapy - surgery, radiation or pharmacotherapy) in the Institute׳s Data Bank and Bio-Repository were included. Subjects were excluded if they had prior cancer history or unclear date of diagnosis, incomplete clinical records, type 1 or unclear diabetes status or history of gestational diabetes. For a specific breakdown of excluded subjects, please see the original research article by Wintrob et al. [1].

A total of 97 female subjects with breast cancer and baseline diabetes mellitus were eligible for inclusion in this analysis.

### 2.3 Control-matching approach

Each of the 97 adult female subjects with breast cancer and diabetes mellitus (defined as “cases”) was matched with two other female subjects diagnosed with breast cancer, but without baseline diabetes mellitus (defined as “controls”). The following matching criteria were used: age at diagnosis, body mass index category, ethnicity, menopausal status and tumor stage (as per the American Joint Committee on Cancer). Some matching limitations applied [1].

### 2.4 Demographic and clinical data collection

Clinical and treatment history was documented as previously described [1]. Vital status was obtained from the Institute׳s Tumor Registry, a database updated biannually with data obtained from the National Comprehensive Cancer Networks’ Oncology Outcomes Database. Outcomes of interest were breast cancer recurrence and/or death.

### 2.5 Plasma specimen storage and retrieval

All the plasma specimens retrieved from long-term storage were individually aliquoted in color coded vials labeled with unique, subject specific barcodes. Overall duration of freezing time was accounted for all matched controls ensuring that the case and matched control specimens had similar overall storage conditions. Only two instances of freeze-thaw were allowed between biobank retrieval and biomarker analyses: aliquoting procedure step and actual assay [1].

### 2.6 Luminex® assays

The following C–C chemokine ligands were quantified according to the manufacturer protocol: chemokine ligand 2, CCL-2 (monocyte chemoattractant protein 1, MCP-1); chemokine ligand 3, CCL-3 (macrophage inflammatory protein 1α, MIP-1α); chemokine ligand 4, CCL-4 (macrophage inflammatory protein 1β, MIP-1β); and chemokine ligand 5, CCL-5 (regulated on activation normal T cell expressed and secreted, RANTES). The HCYTOMAG-60K Luminex® biomarker panel (Millipore Corporation, Billerica, MA) was utilized in this study. Adiponectin, leptin, C-reactive protein, C-peptide, tumor necrosis factor α, interleukine 1β, interleukine 1β receptor antagonist, interleukine 6, and interleukine 10 determinations were done according to the manufacturer protocol as reported [1], [2].

### 2.7 Biomarker-pharmacotherapy association analysis

Biomarker cut-point optimization was performed for each analyzed biomarker. Biomarker levels constituted the continuous independent variable that was subdivided into two groups that optimized the log rank test among all possible cut-point selections yielding a minimum of 10 patients in any resulting group. Quartiles were also constructed. The resultant biomarker categories were then tested for association with type 2 diabetes mellitus therapy and controls by Fisher׳s exact test. The continuous biomarker levels were also tested for association with diabetes therapy and controls across groups by the Kruskall-Wallis test and pairwise by the Wilcoxon rank sum. Multivariate adjustments were performed accounting for age, tumor stage, body mass index, estrogen receptor status, and cumulative comorbidity. The biomarker analysis was performed using R Version 2.15.3. Please see the original article for an illustration of the analysis workflow [1].

Correlations between biomarkers stratified by type 2 diabetes mellitus pharmacotherapy and controls were assessed by the Pearson method. Correlation models were constructed both with and without adjustment for age, body mass index, and the combined comorbidity index. Correlation analyses were performed using SAS Version 9.4.

## Funding sources

This research was funded by the Wadsworth Foundation Peter Rowley Breast Cancer Grant awarded to A.C.C. (UB Grant number 55705, Contract CO26588).

## Figures and Tables

**Table 1:** Pro-inflammatory C–C Chemokine Associations with Insulin Use.

Biomarker	Biomarker Grouping	Concentration	Control	No Insulin	Any Insulin	Unadjusted p-value (MVP)			
						p^1^	p^2^	p^3^	Global Test
CCL-2 (MCP-1, pg/ml)	Median (25th–75th)	–	304 (221–392)	288 (247–402)	320 (207–379)	0.880 (0.740)	0.950 (0.460)	0.990 (0.200)	0.990 (0.480)
								
	Quartiles	1.6 to 225.6	52 (26.9%)	15 (19.7%)	6 (30.0%)	0.090	0.450	0.047	0.100
		227.7 to 302.5	42 (21.8%)	27 (35.5%)	2 (10.0%)				
		303.7 to 388.6	50 (25.9%)	14 (18.4%)	8 (40.0%)				
		391.9 to 4531.2	49 (25.4%)	20 (26.3%)	4 (20.0%)				
								
	OS-Based Optimization	**1.6 to 395.8**^a^	146 (75.6%)	56 (73.7%)	17 (85.0%)	0.740 (0.870)	0.420 (0.250)	0.390 (0.170)	0.600 (0.460)
		398.5 to 4531.2	47 (24.4%)	20 (26.3%)	3 (15.0%)				
								
	DFS-Based Optimization	1.6 to 170.4	22 (11.4%)	6 (7.9%)	3 (15.0%)	0.400 (0.110)	0.710 (0.840)	0.390 (0.360)	0.530 (0.300)
		**172.4 to 4531.2**	171 (88.6%)	70 (92.1%)	17 (85.0%)				
									
CCL-3 (MIP-1α, ng/ml)	Median (25th–75th)	–	3.82 (2.38–6.95)	4.46 (2.38–10.32)	5.49 (2.36–7.58)	0.160 (0.320)	0.580 (0.830)	0.640 (0.520)	0.350 (0.520)
								
	Quartiles	0.36 to 2.37	49 (25.3%)	19 (25.0%)	5 (25.0%)	0.039	0.520	0.120	0.080
		2.41 to 4.02	53 (27.3%)	17 (22.4%)	3 (15.0%)				
		4.07 to 7.96	51 (26.3%)	12 (15.8%)	8 (40.0%)				
		8.11 to 390.27	41 (21.1%)	28 (36.8%)	4 (20.0%)				
								
	OS-Based Optimization	0.36 to 4.02	102 (52.6%)	36 (47.4%)	8 (40.0%)	0.440 (0.280)	0.290 (0.220)	0.560 (0.530)	0.470 (0.290)
		**4.07 to 390.27**^a^	92 (47.4%)	40 (52.6%)	12 (60.0%)				
								
	DFS-Based Optimization	0.36 to 4.02	102 (52.6%)	36 (47.4%)	8 (40.0%)	0.440 (0.280)	0.290 (0.220)	0.560 (0.530)	0.470 (0.290)
		**4.07 to 390.27**	92 (47.4%)	40 (52.6%)	12 (60.0%)				
									
CCL-4 (MIP-1β, pg/ml)	Median (25th–75th)	–	23.00 (16.54–32.87)	27.28 (20.13–42.44)	29.54 (24.27–38.84)	0.017 (0.007)	0.013 (0.230)	0.380 (0.870)	0.006 (0.019)
								
	Quartiles	1.60 to 17.56	56 (28.9%)	14 (18.4%)	2 (10.0%)	0.160	0.100	0.270	0.090
		17.58 to 23.77	48 (24.7%)	22 (28.9%)	3 (15.0%)				
		23.92 to 34.81	48 (24.7%)	16 (21.1%)	8 (40.0%)				
		34.94 to 660.94	42 (21.6%)	24 (31.6%)	7 (35.0%)				
								
	OS-Based Optimization	1.60 to 12.40	18 (9.3%)	4 (5.3%)	1 (5.0%)	0.280 (0.120)	1.000 (0.280)	1.000 (0.970)	0.620 (0.270)
		**12.58 to 660.94**	176 (90.7%)	72 (94.7%)	19 (95.0%)				
								
	DFS-Based Optimization	1.60 to 13.59	26 (13.4%)	5 (6.6%)	1 (5.0%)	0.120 (0.120)	0.480 (0.290)	1.000 (0.760)	0.220 (0.230)
		**13.69 to 660.94**	168 (86.6%)	71 (93.4%)	19 (95.0%)				
									
CCL-5 (RANTES, pg/ml)	Median (25th–75th)	–	7158 (3460–14543)	5958 (3279–9715)	5594 (4386–8821)	0.240 (0.530)	0.430 (0.390)	0.960 (0.650)	0.420 (0.660)
								
	Quartiles	0 to 3446	49 (25.3%)	21 (27.6%)	2 (10.0%)	0.410	0.009	0.110	0.026
		3500 to 6307	41 (21.1%)	21 (27.6%)	11 (55.0%)				
		6381 to 13442	48 (24.7%)	19 (25.0%)	5 (25.0%)				
		13442 to 57898	56 (28.9%)	15 (19.7%)	2 (10.0%)				
								
	OS-Based Optimization	0 to 3183	42 (21.6%)	16 (21.1%)	2 (10.0%)	0.910 (0.920)	0.380 (0.260)	0.350 (0.190)	0.550 (0.380)
		**3212 to 57898**^a^	152 (78.4%)	60 (78.9%)	18 (90.0%)				
								
	DFS-Based Optimization	0 to 16821	160 (82.5%)	69 (90.8%)	19 (95.0%)	0.090 (0.060)	0.210 (0.080)	1.000 (0.570)	0.110 (0.080)
		**16982 to 57898**	34 (17.5%)	7 (9.2%)	1 (5.0%)				

a Overall survival (OS)- and disease-free survival (DFS)-optimized biomarker ranges associated with poorer outcomes are represented in bold. Unadjusted p-values: p1, compares *no insulin versus control*; p2, compares *any insulin versus control*; p3, compares *any insulin versus no insulin* (as per Kruskal-Wallis test); global test, compares *all categories* (as per Wilcoxon, type 3 error test); MVP, denotes the p-value of each multivariate adjusted analysis corresponding to the earlier described unadjusted analyses. For more information, please see Section 2.7 below and our previously published analysis work flow^1^. MVP= p-value of the multivariate adjusted analysis. Chemokine ligand 2, CCL-2 (monocyte chemoattractant protein 1, MCP-1); chemokine ligand 3, CCL-3 (macrophage inflammatory protein 1α, MIP-1α); chemokine ligand 4, CCL-4 (macrophage inflammatory protein 1β, MIP-1β); chemokine ligand 5, CCL-5 (regulated on activation normal T cell expressed and secreted, RANTES).

**Table 2:** Pro-inflammatory Cytokine Correlations by Insulin Use.

Compared Biomarkers			Unadjusted Correlation			Adjusted Correlation		
		Group	Pearson Correlation	95% Confidence Interval	*p*-value	Pearson Correlation	95% Confidence Interval	*p*-value
CCL-2 (MCP-1)	CCL-3 (MIP-1α)	All Subjects (*n*=291)	−0.042	−0.156 to 0.074	0.480	−0.043	−0.158 to 0.073	0.463
		Controls (*n*=194)	−0.034	−0.174 to 0.108	0.636	−0.029	−0.170 to 0.114	0.695
		No Insulin (*n*=77)	−0.140	−0.353 to 0.086	0.221	−0.161	−0.376 to 0.070	0.167
		Any Insulin (*n*=20)	0.063	−0.390 to 0.492	0.788	0.010	−0.473 to 0.489	0.968
CCL-2 (MCP-1)	CCL-4 (MIP-1β)	All Subjects (*n*=291)	0.008	−0.107 to 0.123	0.897	0.008	−0.108 to 0.123	0.892
		Controls (*n*=194)	−0.002	−0.143 to 0.139	0.974	−0.001	−0.143 to 0.141	0.990
		No Insulin (*n*=77)	0.043	−0.183 to 0.264	0.712	0.026	−0.204 to 0.253	0.828
		Any Insulin (*n*=20)	0.065	−0.389 to 0.493	0.784	0.121	−0.382 to 0.568	0.640
CCL-2 (MCP-1)	CCL-5 (RANTES)	All Subjects (*n*=291)	**−0.172**	**−0.281 to −0.058**	**0.003**	**−0.174**	**−0.283 to −0.059**	**0.003**
		Controls (*n*=194)	**−0.257**	**−0.384 to −0.121**	**<0.001**	**−0.251**	**−0.379 to −0.113**	**<0.001**
		No Insulin (*n*=77)	0.057	−0.169 to 0.277	0.622	0.031	−0.199 to 0.257	0.795
		Any Insulin (*n*=20)	−0.144	−0.551 to 0.319	0.539	−0.101	−0.555 to 0.399	0.694
CCL-2 (MCP-1)	IL-1β	All Subjects (*n*=291)	−0.037	−0.151 to 0.078	0.529	−0.036	−0.151 to 0.080	0.545
		Controls (*n*=194)	−0.008	−0.148 to 0.133	0.916	−0.016	−0.158 to 0.126	0.821
		No Insulin (*n*=77)	−0.058	−0.279 to 0.168	0.614	−0.075	−0.299 to 0.156	0.522
		Any Insulin (*n*=20)	−0.017	−0.456 to 0.429	0.944	0.021	−0.464 to 0.497	0.936
CCL-2 (MCP-1)	IL-1Ra	All Subjects (*n*=291)	−0.014	−0.129 to 0.101	0.815	−0.011	−0.127 to 0.104	0.849
		Controls (*n*=194)	−0.007	−0.148 to 0.134	0.923	−0.004	−0.146 to 0.138	0.953
		No Insulin (*n*=77)	−0.019	−0.242 to 0.206	0.867	−0.038	−0.264 to 0.192	0.749
		Any Insulin (*n*=20)	0.036	−0.413 to 0.471	0.879	0.103	−0.397 to 0.556	0.689
CCL-2 (MCP-1)	TNF-α	All Subjects (*n*=291)	−0.013	−0.128 to 0.102	0.824	−0.008	−0.123 to 0.108	0.899
		Controls (*n*=194)	−0.001	−0.142 to 0.140	0.987	−0.018	−0.159 to 0.125	0.808
		No Insulin (*n*=77)	−0.010	−0.234 to 0.214	0.929	0.004	−0.224 to 0.233	0.970
		Any Insulin (*n*=20)	0.098	−0.360 to 0.518	0.677	0.201	−0.309 to 0.622	0.431
CCL-2 (MCP-1)	IL-6	All Subjects (*n*=291)	0.010	−0.105 to 0.124	0.870	0.007	−0.109 to 0.122	0.910
		Controls (*n*=194)	0.015	−0.126 to 0.156	0.831	0.016	−0.126 to 0.158	0.825
		No Insulin (*n*=77)	−0.030	−0.252 to 0.195	0.794	−0.043	−0.269 to 0.187	0.713
		Any Insulin (*n*=20)	0.066	−0.494 to 0.388	0.779	0.054	−0.438 to 0.521	0.834
CCL-2 (MCP-1)	IL-10	All Subjects (*n*=291)	**0.482**	**0.389 to 0.566**	**<0.001**	−00.007	−0.123 to 0.109	0.904
		Controls (*n*=194)	**0.480**	**0.364 to 0.582**	**<0.001**	0.010	−0.132 to 0.152	0.891
		No Insulin (*n*=77)	**0.506**	**0.319 to 0.656**	**<0.001**	−0.042	−0.268 to 0.188	0.722
		Any Insulin (*n*=20)	**0.474**	**0.039 to 0.757**	**0.030**	0.019	−0.466 to 0.495	0.940
CCL-2 (MCP-1)	Adiponectin	All Subjects (*n*=291)	−0.033	−0.083 to 0.147	0.578	0.011	−0.105 to 0.126	0.852
		Controls (*n*=194)	0.032	−0.109 to 0.172	0.656	−0.006	−0.148 to 0.136	0.930
		No Insulin (*n*=77)	0.054	−0.172 to 0.275	0.641	0.076	−0.155 to 0.300	0.517
		Any Insulin (*n*=20)	−0.195	−0.587 to 0.271	0.404	−0.242	−0.647 to 0.270	0.341
CCL-2 (MCP-1)	Leptin	All Subjects (*n*=291)	0.036	−0.079 to 0.151	0.537	0.059	−0.057 to 0.174	0.314
		Controls (*n*=194)	0.006	−0.135 to 0.146	0.937	0.014	−0.128 to 0.156	0.845
		No Insulin (*n*=77)	0.162	−0.064 to 0.373	0.157	0.195	−0.035 to 0.406	0.093
		Any Insulin (*n*=20)	0.016	−0.430 to 0.455	0.948	0.048	−0.443 to 0.517	0.853
CCL-2 (MCP-1)	CRP	All Subjects (*n*=291)	0.000	−0.115 to 0.115	0.996	0.025	−0.091 to 0.140	0.672
		Controls (*n*=194)	−0.009	−0.150 to 0.132	0.901	0.014	−0.128 to −0.156	0.847
		No Insulin (*n*=77)	0.090	−0.136 to 0.308	0.433	0.076	−0.155 to 0.299	0.518
		Any Insulin (*n*=20)	−0.046	−0.478 to 0.405	0.847	−0.041	−0.511 to 0.449	0.876
CCL-2 (MCP-1)	C-Peptide	All Subjects (*n*=291)	0.057	−0.059 to 0.171	0.334	0.074	−0.042 to 0.188	0.212
		Controls (*n*=194)	0.123	−0.018 to 0.259	0.087	0.119	−0.023 to 0.257	0.100
		No Insulin (*n*=77)	−0.086	−0.304 to 0.141	0.456	−0.076	−0.300 to 0.155	0.516
		Any Insulin (*n*=20)	0.005	−0.439 to 0.446	0.985	−0.016	−0.493 to 0.468	0.949
CCL-3 (MIP-1α)	CCL-4 (MIP-1β)	All Subjects (*n*=291)	**0.267**	**0.157 to 0.371**	**<0.001**	**0.268**	**0.157 to 0.372**	**<0.001**
		Controls (*n*=194)	**0.239**	**0.102 to 0.368**	**<0.001**	**0.235**	**0.097 to 0.365**	**0.001**
		No Insulin (*n*=77)	**0.607**	**0.443 to 0.732**	**<0.001**	**0.601**	**0.431 to 0.729**	**<0.001**
		Any Insulin (*n*=20)	**0.523**	**0.105 to 0.784**	**0.014**	**0.700**	**0.330 to 0.883**	**<0.001**
CCL-3 (MIP-1α)	CCL-5 (RANTES)	All Subjects (*n*=291)	0.091	−0.025 to 0.204	0.122	0.092	−0.024 to 0.205	0.119
		Controls (*n*=194)	0.107	−0.035 to 0.244	0.138	0.108	−0.034 to 0.247	0.134
		No Insulin (*n*=77)	−0.033	−0.255 to 0.192	0.773	−0.055	−0.280 to 0.175	0.638
		Any Insulin (*n*=20)	0.120	−0.341 to 0.534	0.610	0.068	−0.427 to 0.531	0.794
CCL-3 (MIP-1α)	IL-1β	All Subjects (*n*=291)	**0.151**	**0.037 to 0.261**	**<0.010**	**0.156**	**0.041 to 0.267**	**0.008**
		Controls (*n*=194)	0.092	−0.050 to 0.229	0.203	0.092	−0.051 to 0.231	0.205
		No Insulin (*n*=77)	**0.561**	**0.386 to 0.698**	**<0.001**	**0.560**	**0.380 to 0.699**	**<0.001**
		Any Insulin (*n*=20)	**0.470**	**0.034 to 0.755**	**0.031**	**0.610**	**0.184 to 0.844**	**0.006**
CCL-3 (MIP-1α)	IL-1Ra	All Subjects (*n*=291)	**0.232**	**0.120 to 0.338**	**<0.001**	**0.232**	**0.120 to 0.339**	**<0.001**
		Controls (*n*=194)	**0.223**	**0.085 to 0.353**	**0.002**	**0.215**	**0.076 to 0.347**	**0.003**
		No Insulin (*n*=77)	**0.511**	**0.325 to 0.660**	**<0.001**	**0.510**	**0.319 to 0.662**	**<0.001**
		Any Insulin (*n*=20)	0.370	−0.086 to 0.698	0.100	**0.604**	**0.174 to 0.841**	**0.007**
CCL-3 (MIP-1α)	TNF-α	All Subjects (*n*=291)	**0.163**	**0.049 to 0.273**	**0.005**	**0.170**	**0.055 to 0.280**	**0.004**
		Controls (*n*=194)	0.112	−0.030 to 0.249	0.120	0.110	−0.033 to 0.248	0.129
		No Insulin (*n*=77)	**0.570**	**0.397 to 0.704**	**<0.001**	**0.585**	**0.412 to 0.718**	**<0.001**
		Any Insulin (*n*=20)	0.389	−0.065 to 0.709	0.083	**0.639**	**0.229 to 0.857**	**0.004**
CCL-3 (MIP-1α)	IL-6	All Subjects (*n*=291)	0.106	−0.009 to 0.219	0.070	0.110	−0.006 to 0.223	0.062
		Controls (*n*=194)	0.092	−0.050 to 0.230	0.202	0.101	−0.042 to 0.239	0.165
		No Insulin (*n*=77)	**0.353**	**0.140 to 0.535**	**<0.002**	**0.337**	**0.118 to 0.525**	**0.003**
		Any Insulin (*n*=20)	0.249	−0.217 to 0.623	0.281	**0.560**	**0.109 to 0.820**	**0.015**
CCL-3 (MIP-1α)	IL-10	All Subjects (*n*=291)	**0.164**	**0.050 to 0.274**	**0.005**	**0.163**	**0.049 to 0.274**	**0.005**
		Controls (*n*=194)	**0.201**	**0.062 to 0.332**	**<0.005**	**0.195**	**0.055 to 0.328**	**0.006**
		No Insulin (*n*=77)	**0.312**	**0.095 to 0.501**	**0.005**	**0.308**	**0.086 to 0.502**	**0.007**
		Any Insulin (*n*=20)	**0.661**	**0.309 to 0.854**	**<0.001**	**0.543**	**0.085 to 0.812**	**0.019**
CCL-3 (MIP-1α)	Adiponectin	All Subjects (*n*=291)	−0.058	−0.172 to 0.057	0.324	−0.051	−0.166 to 0.065	0.388
		Controls (*n*=194)	−0.078	−0.217 to 0.063	0.277	−0.049	−0.189 to 0.094	0.502
		No Insulin (*n*=77)	−0.018	−0.241 to 0.207	0.876	−0.032	−0.259 to 0.197	0.783
		Any Insulin (*n*=20)	0.308	−0.155 to 0.661	0.178	0.169	−0.339 to 0.601	0.510
CCL-3 (MIP-1α)	Leptin	All Subjects (*n*=291)	0.052	−0.063 to 0.166	0.374	0.029	−0.087 to 0.144	0.622
		Controls (*n*=194)	0.073	−0.068 to 0.212	0.309	0.029	−0.114 to 0.170	0.692
		No Insulin (*n*=77)	−0.001	−0.225 to 0.223	0.996	0.018	−0.211 to 0.246	0.877
		Any Insulin (*n*=20)	−0.112	−0.528 to 0.348	0.634	0.133	−0.372 to 0.577	0.606
CCL-3 (MIP-1α)	CRP	All Subjects (*n*=291)	0.036	−0.079 to 0.150	0.539	0.017	−0.098 to 0.133	0.769
		Controls (*n*=194)	0.053	−0.088 to 0.193	0.460	0.100	−0.132 to 0.152	0.892
		No Insulin (*n*=77)	0.075	−0.152 to 0.294	0.517	0.079	−0.152 to 0.302	0.501
		Any Insulin (*n*=20)	−0.194	−0.586 to 0.272	0.406	−0.035	−0.507 to 0.453	0.891
CCL-3 (MIP-1α)	C-Peptide	All Subjects (*n*=291)	−0.038	−0.153 to 0.077	0.515	−0.045	−0.160 to 0.071	0.446
		Controls (*n*=194)	−0.023	−0.163 to 0.119	0.753	−0.034	−0.175 to 0.109	0.644
		No Insulin (*n*=77)	−0.147	−0.359 to 0.080	0.200	−0.130	−0.348 to 0.102	0.269
		Any Insulin (*n*=20)	−0.306	−0.659 to 0.158	0.181	−0.235	−0.643 to 0.277	0.354
CCL-4 (MIP-1β)	CCL-5 (RANTES)	All Subjects (*n*=291)	−0.009	−0.124 to 0.106	0.872	−0.008	−0.123 to 0.108	0.894
		Controls (*n*=194)	−0.039	−0.179 to 0.102	0.588	−0.038	−0.179 to 0.105	0.601
		No Insulin (*n*=77)	0.083	−0.144 to 0.301	0.471	0.058	−0.173 to 0.283	0.622
		Any Insulin (*n*=20)	0.105	−0.354 to 0.523	0.655	0.056	−0.436 to 0.523	0.828
CCL-4 (MIP-1β)	IL-1β	All Subjects (*n*=291)	**0.574**	**0.491 to 0.646**	**<0.001**	**0.574**	**0.491 to 0.647**	**<0.001**
		Controls (*n*=194)	**0.217**	**0.079 to 0.347**	**0.002**	**0.217**	**0.078 to 0.348**	**0.002**
		No Insulin (*n*=77)	**0.851**	**0.775 to 0.903**	**<0.001**	**0.849**	**0.770 to 0.903**	**<0.001**
		Any Insulin (*n*=20)	**0.829**	**0.611 to 0.930**	**<0.001**	**0.809**	**0.538 to 0.929**	**<0.001**
CCL-4 (MIP-1β)	IL-1Ra	All Subjects (*n*=291)	**0.836**	**0.798 to 0.868**	**<0.001**	**0.836**	**0.798 to 0.868**	**<0.001**
		Controls (*n*=194)	**0.875**	**0.838 to 0.905**	**<0.001**	**0.875**	**0.838 to 0.905**	**<0.001**
		No Insulin (*n*=77)	**0.807**	**0.711 to 0.873**	**<0.001**	**0.807**	**0.710 to 0.874**	**<0.001**
		Any Insulin (*n*=20)	**0.914**	**0.791 to 0.966**	**<0.001**	**0.918**	**0.782 to 0.970**	**<0.001**
CCL-4 (MIP-1β)	TNF-α	All Subjects (*n*=291)	**0.438**	**0.340 to 0.527**	**<0.001**	**0.446**	**0.349 to 0.534**	**<0.001**
		Controls (*n*=194)	**0.421**	**0.298 to 0.531**	**<0.001**	**0.430**	**0.307 to 0.539**	**<0.001**
		No Insulin (*n*=77)	**0.422**	**0.219 to 0.590**	**<0.001**	**0.448**	**0.245 to 0.614**	**<0.001**
		Any Insulin (*n*=20)	**0.829**	**0.610 to 0.930**	**<0.001**	**0.805**	**0.529 to 0.927**	**<0.001**
CCL-4 (MIP-1β)	IL-6	All Subjects (*n*=291)	**0.334**	**0.228 to 0.433**	**<0.001**	**0.336**	**0.230 to 0.435**	**<0.001**
		Controls (*n*=194)	**0.317**	**0.184 to 0.438**	**<0.001**	**0.322**	**0.188 to 0.443**	**<0.001**
		No Insulin (*n*=77)	**0.647**	**0.495 to 0.761**	**<0.001**	**0.646**	**0.489 to 0.762**	**<0.001**
		Any Insulin (*n*=20)	**0.853**	**0.660 to 0.941**	**<0.001**	**0.884**	**0.700 to 0.958**	**<0.001**
CCL-4 (MIP-1β)	IL-10	All Subjects (*n*=291)	**0.701**	**0.637 to 0.755**	**<0.001**	**0.702**	**0.638 to 0.756**	**<0.001**
		Controls (*n*=194)	**0.726**	**0.652 to 0.787**	**<0.001**	**0.726**	**0.651 to 0.787**	**<0.001**
		No Insulin (*n*=77)	**0.770**	**0.660 to 0.848**	**<0.001**	**0.770**	**0.657 to 0.849**	**<0.001**
		Any Insulin (*n*=20)	0.301	−0.163 to 0.656	0.188	0.364	−0.141 to 0.719	0.141
CCL-4 (MIP-1β)	Adiponectin	All Subjects (*n*=291)	−0.023	−0.137 to 0.092	0.698	−0.026	−0.142 to 0.089	0.655
		Controls (*n*=194)	−0.002	−0.143 to 0.139	0.974	0.011	−0.131 to 0.153	0.879
		No Insulin (*n*=77)	−0.051	−0.272 to 0.175	0.657	−0.065	−0.289 to 0.166	0.583
		Any Insulin (*n*=20)	0.181	−0.285 to 0.577	0.439	0.207	−0.304 to 0.625	0.418
CCL-4 (MIP-1β)	Leptin	All Subjects (*n*=291)	−0.038	−0.152 to 0.077	0.518	−0.049	−0.163 to 0.067	0.411
		Controls (*n*=194)	−0.017	−0.158 to 0.124	0.811	−0.043	−0.184 to 0.100	0.556
		No Insulin (*n*=77)	−0.073	−0.293 to 0.153	0.524	0.004	−0.224 to 0.233	0.970
		Any Insulin (*n*=20)	−0.217	−0.602 to 0.249	0.350	−0.060	−0.525 to 0.434	0.819
CCL-4 (MIP-1β)	CRP	All Subjects (*n*=291)	0.096	−0.019 to 0.209	0.102	0.102	−0.013 to 0.215	0.082
		Controls (*n*=194)	**0.195**	**0.056 to 0.327**	**0.006**	**0.198**	**0.057 to 0.330**	**0.006**
		No Insulin (*n*=77)	−0.017	−0.240 to 0.208	0.884	0.015	−0.214 to 0.242	0.900
		Any Insulin (*n*=20)	−0.268	−0.635 to 0.198	0.245	−0.173	−0.604 to 0.335	0.499
CCL-4 (MIP-1β)	C-Peptide	All Subjects (*n*=291)	−0.098	−0.210 to 0.018	0.096	−0.105	−0.218 to 0.011	0.076
		Controls (*n*=194)	−0.116	−0.253 to 0.025	0.106	−0.123	−0.261 to 0.019	0.089
		No Insulin (*n*=77)	−0.121	−0.336 to 0.106	0.293	−0.077	−0.301 to 0.154	0.511
		Any Insulin (*n*=20)	−0.426	−0.731 to 0.020	0.054	−0.351	−0.711 to 0.156	0.158
CCL-5 (RANTES)	IL-1β	All Subjects (*n*=291)	0.037	−0.079 to 0.151	0.535	0.040	−0.076 to 0.155	0.500
		Controls (*n*=194)	0.081	−0.060 to 0.220	0.258	0.088	−0.055 to 0.227	0.225
		No Insulin (*n*=77)	0.061	−0.165 to 0.281	0.596	0.040	−0.191 to 0.266	0.737
		Any Insulin (*n*=20)	−0.012	−0.452 to 0.433	0.959	−0.080	−0.540 to 0.417	0.757
CCL-5 (RANTES)	IL-1Ra	All Subjects (*n*=291)	0.008	−0.107 to 0.123	0.895	0.008	−0.107 to 0.124	0.888
		Controls (*n*=194)	0.011	−0.130 to 0.152	0.874	0.013	−0.129 to 0.155	0.857
		No Insulin (*n*=77)	0.025	−0.200 to 0.248	0.827	0.002	−0.226 to 0.231	0.985
		Any Insulin (*n*=20)	−0.007	−0.448 to 0.437	0.977	−0.045	−0.514 to 0.446	0.863
CCL-5 (RANTES)	TNF-α	All Subjects (*n*=291)	−0.064	−0.178 to 0.051	0.274	−0.047	−0.162 to 0.069	0.422
		Controls (*n*=194)	−**0.146**	−**0.281 to −0.005**	**0.042**	−**0.143**	−**0.279 to −0.001**	**0.048**
		No Insulin (*n*=77)	0.059	−0.168 to 0.279	0.611	0.080	−0.151 to 0.303	0.497
		Any Insulin (*n*=20)	0.201	−0.265 to 0.591	0.388	0.169	−0.339 to 0.601	0.510
CCL-5 (RANTES)	IL-6	All Subjects (*n*=291)	0.051	−0.065 to 0.165	0.388	0.047	−0.069 to 0.161	0.430
		Controls (*n*=194)	0.043	−0.098 to 0.183	0.546	0.042	−0.100 to 0.183	0.562
		No Insulin (*n*=77)	0.046	−0.180 to 0.267	0692	0.032	−0.198 to 0.258	0.788
		Any Insulin (*n*=20)	0.216	−0.251 to 0.601	0.354	0.124	−0.379 to 0.571	0.631
CCL-5 (RANTES)	IL-10	All Subjects (*n*=291)	0.025	−0.090 to 0.140	0.666	0.023	−0.093 to 0.138	0.700
		Controls (*n*=194)	0.013	−0.128 to 0.154	0.857	0.016	−0.126 to 0.158	0.824
		No Insulin (*n*=77)	0.058	−0.168 to 0.279	0.612	0.036	−0.194 to 0.262	0.762
		Any Insulin (*n*=20)	−0.004	−0.446 to 0.439	0.986	−0.076	−0.537 to 0.420	0.769
CCL-5 (RANTES)	Adiponectin	All Subjects (*n*=291)	0.014	−0.101 to 0.129	0.816	0.022	−0.094 to 0.137	0.713
		Controls (*n*=194)	0.022	−0.119 to 0.163	0.757	0.038	−0.105 to 0.179	0.603
		No Insulin (*n*=77)	−0.132	−0.346 to 0.095	0.250	−0.120	−0.339 to 0.112	0.307
		Any Insulin (*n*=20)	0.146	−0.317 to 0.553	0.533	0.108	−0.393 to 0.560	0.676
CCL-5 (RANTES)	Leptin	All Subjects (*n*=291)	−0.037	−0.151 to 0.078	0.528	−0.016	−0.131 to 0.100	0.788
		Controls (*n*=194)	−0.068	−0.207 to 0.073	0.344	−0.073	−0.212 to 0.070	0.318
		No Insulin (*n*=77)	0.050	−0.176 to 0.271	0.665	0.094	−0.138 to 0.315	0.426
		Any Insulin (*n*=20)	0.229	−0.238 to 0.610	0.324	0.444	−0.046 to 0.762	0.066
CCL-5 (RANTES)	CRP	All Subjects (*n*=291)	−0.083	−0.196 to 0.032	0.157	−0.074	−0.188 to 0.042	0.207
		Controls (*n*=194)	−0.077	−0.216 to 0.065	0.285	−0.100	−0.237 to 0.045	0.177
		No Insulin (*n*=77)	−0.116	−0.332 to 0.111	0.312	−0.131	−0.349 to 0.100	0.263
		Any Insulin (*n*=20)	0.260	−0.206 to 0.630	0.259	0.421	−0.075 to 0.750	0.084
CCL-5 (RANTES)	C-Peptide	All Subjects (*n*=291)	−0.028	−0.143 to 0.087	0.634	−0.013	−0.128 to 0.103	0.832
		Controls (*n*=194)	−0.014	−0.155 to 0.127	0.843	−0.012	−0.154 to 0.130	0.868
		No Insulin (*n*=77)	−0.012	−0.235 to 0.213	0.918	0.015	−0.214 to 0.243	0.897
		Any Insulin (*n*=20)	−0.019	−0.458 to 0.427	0.935	0.108	−0.393 to 0.559	0.677

Significant correlations are displayed in bolded text. The differences that are only significant in either adjusted or unadjusted correlations are further denoted by an outline. Chemokine ligand 2, CCL-2 (monocyte chemoattractant protein 1, MCP-1); chemokine ligand 3, CCL-3 (macrophage inflammatory protein 1α, MIP-1α); chemokine ligand 4, CCL-4 (macrophage inflammatory protein 1β, MIP-1β); chemokine ligand 5, CCL-5 (regulated on activation normal T cell expressed and secreted, RANTES); adiponectin; leptin; C-reactive protein, CRP; C-peptide; tumor necrosis factor α, TNF-α; interleukine 1β, IL-1β; interleukine 1β receptor antagonist, IL-1Ra; interleukine 6, IL-6; and interleukine 10, IL-10.
